# The Galveston County Medical Society

**Published:** 1892-12

**Authors:** 


### Society Notes.

### THE GALVESTON COUNTY MEDICAL SOCIETY.

The Faculty of the Medical Department of the University of Texas, having obtained permission from the resident Regent until such time when the Regents in session should take further action, appointed a committee of three of its members to issue an invitation to the members of the medical profession of Galveston county to convene on October 31, 1892, in the Medical Hall, for the purpose of organizing a County Medical Society. At this meeting, some twenty gentlemen being present, Dr. Randall, as Chairman of the Committee from the Faculty, presided. After due consideration of a motion to proceed to the organization of a Medical Association, with the usual objects of such a body, it was determined by vote of those present that such a society should be created, and a committee, composed of Drs. Allen J. Smith, H. P. Cooke and R. C. Hodges was named to draft a suitable set of rules for the conduct of the same. At an adjourned meeting, held on November 4, 1892, this committee reported the constitution and by-laws prepared in the interval, which were accepted. The election of officers was then held and the following gentlemen were elected: President, Dr. A. W. Fly; First Vice-President, Dr. W. Keiller; Second Vice-President, Dr. V. H. Hulen; Treasurer, Dr. W. H. Baldinger; Secretary, Dr. S. M. Morris; Executive Committee, Drs. Allen J. Smith, G. P. Hall and Ed. Randall. The meetings of the Society are to be held on the first and third Mondays of each month, at 8 o'clock in the evening, in the Medical Hall of the University of Texas, during the annual term extending from the third Monday in September to the first Monday in May. It is the purpose of the Society to turn a portion of its income to the subscription of journals and purchase of books to be placed in the reading room, and eventually in the library of the medical school, provided it can be arranged that these portions of the department may be open to the use of the profession generally as well as of the students of the college. There have been held two regular meetings, at the first of which Dr. David Cerna read a paper on Belladonna and Opium (see page 205 of the JOURNAL), and at the second, Dr. Keiller presented a beautifully illustrated paper on Axis-Traction Forceps. Cases of interest and specimens of pathological value were exhibited and discussed with enthusiasm by the members; and thus far the liveliest zeal has marked the proceedings of the Association. It is to be earnestly hoped that the friendly relations thus established between the State Medical School and the profession about it will be long continued to the benefit of both. With the present Executive Committee in charge of the publication of the papers presented before the Association, DANIEL'S TEXAS MEDICAL JOURNAL will doubtless be permitted to offer to its readers a large proportion of the Society's work.

